# 

**Published:** 1788

**Authors:** 


### LONDON MEDICAL JOURNAL.

VOLUME THE NINTH.

FOR THE YEAR 1788.

Surgeon General's Office. LIBRARY. 63448 Washington, D.C.

LONDON:

PRINTED for the EDITOR,

And fold by JOSEPH JOHNSON, N°. 72,
ST. PAUL'S CHURCH YARD.

M,DCC,LXXXVIII.

*Filamenta* decem, receptaculo inserta, petalis paulo breviora, inferne connata in cylindrum longitudine calycis, superne infra antheras ciliata. *Antheræ* lineares, filamentis longitudinaliter adnatæ, iisque dimidio breviores. *Germen* superum, ovatum, tomentosum. *Stylus* filiformis, staminibus longior. *Stigma* simplex.

---

### CATALOGUE OF BOOKS.

1. PRINCIPIA Botanica: or a Concise and easy Introduction to the sexual Botany of Linnæus. With the Genera; their mode of growth (as tree, shrub, or herb;) the number of species to each genus; where principally native; and the number indigenous to the British isles: arranged in columns under each class and order; and digested alphabetically under several generical distinctions. By which means most plants may be thus far ascertained. Together with three Indexes. 1. Of the Linnæan genera accented, with the British names. 2. Of such trivial names as were the genera of old authors. 3. Of the British names, with the Linnæan genera; to which are added many many of the ſpecific names; alſo a table of ſeveral vegetable drugs not in the Indexes. 8vo. Newark, 1787.

2. Eſſays on the Hepatitis, and Spaſmodic Affections in India, founded on obſervations made whilſt on ſervice with his Majeſty's troops in different parts of that country. By *Thomas Girdleſtone*, M. D. 8vo. *Murray*, London, 1787.

3. Firſt lines of the Theory and Practice of the Venereal Diſeaſe. By *William Niſbet*, Fellow of the Royal College of Surgeons of Edinburgh. 8vo. Edinburgh, 1787.

4. Specimen Artis Obſtetricariæ: Being a Syllabus, or general Heads of a Courſe of Lectures on the Theory and Practice of Midwifery, and Diſeaſes incident to Women and Children. By *John Leake*, M. D. Member of the Royal College of Phyſicians, and Phyſician to the Weſtminſter Lying-in Hoſpital. 8vo. *Murray*, London, 1787.

5. The Prognoſtics and Prorrhetics of Hippocrates, tranſlated from the original Greek, with large Annotations, critical and explanatory; to which is prefixed a ſhort Account of the Life of Hippocrates. By *John Moffat*, M. D. 8vo. *Elliott*, London, 1787.

6. Conciſe

6. Concise Observations on the Nature of Common Food, so far as it tends to promote or injure health; with remarks on water, bread, meat, cheese, butter, milk, wine, punch, beer, coffee, tea, sugar, &c. By a gentleman of the faculty. 8vo. *Wilkie*, London, 1787.

7. The Case of a Boy, who had been mistaken for a Girl; with three anatomical views of the parts before and after the operation and cure. By *Thomas Brand*, Surgeon. 4to. *Nicol*, London, 1787.

8. A Treatise on Elementary Air. By *Hamilton Kelso*, M. D. 8vo. London, 1787.

9. A Synopsis of a Course of Lectures on Anatomy and Physiology. By *Busick Harwood*, M. B. F. R. S. Professor of Anatomy in the University of Cambridge. 8vo. *Cadell*, London, 1787.

10. Narrative of the Efficacy of the Bath Waters in various kinds of paralytic disorders, admitted into the Bath Hospital from the end of 1775 to the end of 1785, with particular relations of fifty-two of their cases. Published by order of the Committee, at the expence and for the benefit of the Hospital. 8vo. *Cruttwell*, Bath, 1787.

11. Obſervations on Medical Electricity; containing a Synopſis of all the diſeaſes in which Electricity has been recommended or applied with ſucceſs; likewiſe pointing out a new and more efficacious method of applying this remedy by electric vibrations. By *Francis Lowndes*. 8vo. London, 1787.

12. Chemical Eſſays. By *Richard Watſon*, D. D. F. R. S. Lord Biſhop of Landaff, and Regius Profeſſor of Divinity in the Univerſity of Cambridge. Vol. V. 8vo. *Evans*, London, 1787.

13. Obſervations on the Specimen Alterum Pharmacopœiæ Londinenſis, 1787; pointing out its many ſtriking defects, and ſhewing the neceſſity for ſtill farther corrections, in order to aſſiſt in conſtituting a work ſo greatly wanted, in as perfect a ſtate as the difficulty of the undertaking will permit, and the magnitude of the ſubject requires: interſperſed with a variety of formulæ from that publication, and other new ones introduced. In a letter, addreſſed to the Committee ſelected out of the Members of the Royal College of Phyſicians to reform the old Pharmacopœia, a proof of which reformation is preſented in the Specimen. 8vo. *Robinſon*, London, 1787.

14. Prin-

14. Principles of Surgery. Part the Firſt. By *John Pearſon*, Surgeon to the Lock Hoſpital, and to the Public Diſpenſary. 8vo. *Johnſon*, London, 1788.

15. Tranſactions of the Royal Society of Edinburgh. Vol. I. 4to. Edinburgh, 1788.

16. Pharmacopœia Collegii Regalis Medicorum Londinenſis. 4to. et 12mo. *Johnſon*, Londini, 1788.

17. The New Pharmacopœia of the Royal College of Phyſicians of London. Tranſlated into Engliſh, with notes, indexes of new names, preparations, &c. By *Thomas Healde*, M. D. F. R. S. Lumleyan Lecturer at the College of Phyſicians, and Senior Phyſician of the London Hoſpital. 8vo. *Longman*, London, 1788.

18. The Hiſtory of Funguſſes, growing about Halifax: with forty-four copper-plates; on which are engraved fifty-one ſpecies of Agarics: wherein their varieties, and various appearances in the different ſtages of growth, are faithfully exhibited in more than two hundred figures, copied with great care from the plants, when newly gathered and in a ſtate of perfection. With a particular deſcription of each ſpecies, in all its ſtages, from the firſt appearappearance to the utter decay of the plant; with the time when they were gathered; the foil and fituation in which they grew; their duration; and the particular places mentioned, where all the new or rare fpecies were found. The whole being a plain recital of facts, the refult of more than twenty years obfervation. In three volumes. By *James Bolton*, Member of the Nat. Hift. Society at Edinburgh. Vol. I. 4to. *White*, London, 1788.

19. A Treatife on Tropical Difeafes, and on the Climate of the Weft Indies. In which are included, the treatment and cure of the ftings of fcorpions, and of the ftings and bites of other poifonous infects; of the bites of deadly venomous ferpents; of the bites of mad dogs; of the dyfentery; of the yellow fever; of the tetanos, or locked jaw; of cancers; of the belly ach, to which painters, printers, &c. are fubject; of hæmorrhages from the lungs; of the afthma; of catarrhal coughs; of the hooping cough, &c. By *Benjamin Mofeley*, M. D. Member of the Royal College of Phyficians, London. 8vo. *Cadell*, London, 1788.

20. The works of the late *William Stark*, M. D. confifting of Clinical and Anatomical Obfervations, with Experiments Dietetical and

Statical, revifed and publifhed from his original MS. By *James Carmichael Smyth*, M. D. F. R. S. Phyfician Extraordinary to his Majefty. 4to. *Johnfon*, London, 1788.

21. Obfervations on the inefficacious Ufe of Irons in Cafes of Luxations, and Diftortions of the Ancle Joint, and Children born with diftorted and crooked feet; a much more agreeable and effectual mode of treatment being purfued, illuftrated with extraordinary Cures, and addreffed to the Public. By *William Jackfon*, Member of the Univerfity of Oxford, of the Corporation of Surgeons of London, late Surgeon in the Army, and now a Practitioner at Iflington. 8vo. *Symonds*, London, 1788.

22. A Candid Review of Jeffe Foot's Obfervations on the new opinions of John Hunter, in his late Treatife on the Venereal Difeafe, ending with the fubject of Gonorrhœa. By *John Peake*, Surgeon. 8vo. *Johnfon*, London, 1788.

23. Chemical Obfervations on Sugar. By *Edward Rigby*. 8vo. *Johnfon*, London, 1788.

24. Practical Obfervations on Herniæ; illuftrated with Cafes. By *B. Wilmer*, Surgeon in Coventry. 8vo. *Longman*, London, 1788.

25. Method of Chemical Nomenclature, proposed by Messrs. de Morveau, Lavoisier, Bertholet, and de Fourcroy. To which is added a new System of Chemical Characters adapted to the Nomenclature, by Messrs. Hassenfratz and Adet. Translated from the French, and the new Chemical Names adapted to the Genius of the English Language, with the Approbation, and under the immediate Inspection of M. de Fourcroy. By *James St. John*, M. D. 8vo. *Kearsley*, London, 1788.

26. Dissertatio Medica Inauguralis quædam de Puerperarum febre complectens. Auctore *Petro Ashton*, Anglo. 8vo. Edinburgi, 1787.

27. Dissertatio Medica Inauguralis de Nephritide. Auctore *Jacobo Caldwall*, Hiberno. 8vo. Edin. 1787.

28. Dissertatio Physica Inauguralis quædam de Fermentatione, et quibusdam mutationibus inde pendentibus proponens. Auctore *Joanne Carmichaell*, Scoto. 8vo. Edin. 1787.

29. Dissertatio Medica Inauguralis de Rachitide. Auctore *Gulielmo Hamersley*, Reipublicæ Novi-Eboraci Civi. 8vo. Edin. 1787.

30. Dissertatio quædam de Asthmate Spasmodico complectens. Auctore *Joanne Heath*, Anglo. 8vo. Edin. 1787.

31. Tentamen Inaugurale quædam de Plantarum motibus et Vita complectens. Auctore *Thoma Carolo Hope*, Scoto Britanno. 8vo. Edin. 1787.

32. Dissertatio Medica Inauguralis de Ictero. Auctore *Pinkston James*, Londinensi. 8vo. Edin. 1787.

33. Dissertatio Medica Inauguralis de Rheumatismo. Auctore *Andrea Inglis*, Edinensi. 8vo. Edin. 1787.

34. Dissertatio Medica Inauguralis de Rheumatismo acuto. Auctore *Ricardo Sharpe Kissam*, Americano. 8vo. Edin. 1787.

35. Dissertatio Medica Inauguralis de Hæmorrhoide. Auctore *Jacobo Low*, Fifensi. 8vo. Edin. 1787.

36. Dissertatio Medica Inauguralis quædam de Scorbuto colligens. Auctore *Henrico Luxmoore*, Anglo. 8vo. Edin. 1787.

37. Dissertatio Medica Inauguralis de Apoplexia Plethorica. Auctore *Joanne M'Cartney*, Scoto. 8vo. Edin. 1787.

38. Dissertatio Medica Inauguralis de Hepatitide. Auctore *Roberto M'Causland*, Britanno. 8vo. Edin. 1787.

39. Dissertatio Physiologica Inauguralis de Actione Musculari. Auctore *Jacobo Mackintosh*, ex Agro Innernessano, Scoto. 8vo. Edin. 1787.

40. Diſſertatio Inauguralis Experimentorum quorundam, cum diverſis Aerum ſpeciebus in Animalibus inſtitutorum, phenomena exhibens. Auctore *Gulielmo Maxwell*, Scoto. 8vo. Edin. 1787.

41. Diſſertatio Medica Inauguralis de Dyſpepſia. Auctore *Franciſco Moriſon*, Edinburgenſi. 8vo. Edin. 1787.

42. Diſſertatio Medica Inauguralis de Abortu. Auctore *Joanne Rogero Murray*, Britanno. 8vo. Edin. 1787.

43. Diſſertatio Medica Inauguralis de Colica ſpaſmodica. Auctore *Andrea Ponton*, Edinburgenſi. 8vo. Edin. 1787.

44. Tentamen Chymico-Medicum Inaugurale de Calculo Urinario et Lithontripticis. Auctore *Jacobo Reynolds*, Hiberno. 8vo. Edin. 1787.

45. Diſſertatio Medica Inauguralis de Paralyſi. Auctore *Patricio Baron Seton*, Scoto. 8vo. Edin. 1787.

46. Diſſertatio Medica Inauguralis de Morbillis. Auctore *Auguſtino Smith*, Virginienſi. 8vo. Edin. 1787.

47. Diſſertatio Medica Inauguralis de Ophthalmia. Auctore *Joanne Smith*, Americano. 8vo. Edin. 1787.

48. Dissertatio Medica Inauguralis de Erysipelate. Auctore *Roberto Smithwick*, Hiberno. 8vo. Edin. 1787.

49. Dissertatio Medica Inauguralis de Gonorrhœa Virulenta. Auctore *H. Stanistreet*, Anglo. 8vo. Edin. 1787.

50. Dissertatio Medica Inauguralis de Amenorrhœa. Auctore *Alexandro Straith*, ex Agro Narniensi, Scoto. 8vo. Edin. 1787.

51. Dissertatio Medica Inauguralis de Hæmorrhagiis. Auctore *Joanne Taylor*, Cathenesiensi. 8vo. Edin. 1787.

52. Dissertatio Medica Inauguralis de Angina Tonsillari. Auctore *Thoma Wallace*, Hiberno. 8vo. Edin. 1787.

53. *Henrici Augusti Wrisberg*, Phil. et Med. Doct. Consil. Aulici Regii et Electoralis, Med. et Anat. in Univers. Georgia Augusta Prof. p. et o. De Uteri mox post partum resectione non lethali, Commentatio, Observatione illustrata, cum brevissima principiorum lethalitatis Sciagraphia. 4to. Goettingæ. 1787.

54. Amphibiorum virtutis medicatæ Defensio inchoata, quam præside *Johanne Hermann*, Doct. et Prof. Med. Publ. Cap. Thom. Can. Solemniter defendet die 29 Martii A. 1787.

*Johannes*

*Johannes Godofredus Schneiter*, Argentinensis. 4to. Argentorati, 1787.

55. Dissertatio Sistens Observationes et Experimenta circa genesin Aeris fixi et Phlogisticati. Auct. *Fr. A. C. Gren.* 8vo. Halle, 1786.

56. *F. H. Rhades* Animadversiones circa Temperamenta humana, imprimisque ea, quæ Lactatione communicata habentur. 8vo. Halæ, 1787.

57. Commentatio Medico-Obstetricia de Utero reverso. Auctore *Friderico Jahn*, M. D. 8vo. Jena, 1787.

58. *J. D. Schoepf*, Marggr. Onold. et Culmb. Med. Aul. Materia Medica Americana, potissimum Regni Vegetabilis. 8vo. Erlangæ, 1787.

59. *Christ. Frid. Ludwig* Phil. et Med. D. Med. et Hist. Nat. P. P. in Academia Lipsiensi, Soc. Oec. Lips. et Musei Parisini Sodalis, Historiæ Anatomiæ et Physiologiæ comparantis brevis expositio. 4to. Lipsiæ, 1787.

60. Lectiones publicæ de vermibus intestinalibus inprimis Humanis, quas habuit in musæo Rer. Nat. Acad. Lundensis D. 18 Martii et Sequ. 1784, *Anders Jahan Retzius*, Professor R. D. 8vo. Holmiæ, 1786.

61. Dissertatio medica de Phrenitide, quam præside *Jona Sidren*, M. D. Med. Pract. Prof. Reg.

Reg. et Ord. pro gradu Doctoris publico examini subjicit *Johannes Ericus Gevalin*, V. Gothus. 4to. Upsaliæ, 1786.

62. Vasorum Lacteorum atque Lymphaticorum anatomico-physiologica Descriptio. Fasciculus I. Ediderunt *Paullus Christianus Fridericus Werner*, *Christianus Gotthold Feller*. cum Tabb. IV. 4to. Lipsiæ, 1784.

63. Consultation medico-legale sur une Accusation d'Infanticide. Par M. *Chaussier*. 4to. Dijon, 1786.

64. Eloge historique de *Michel Philippe Bouvart*, Chevalier de l'ordre de Saint Michel, Docteur Regent de la Faculté de Medecine en l'Université de Paris, de l'Academie Royale des Sciences, ancien Professeur de Medecine au College Royal de France, &c. Par M. *A. J. B. M. Guenet*, Docteur-Regent de la Faculté de Medecine de Paris, &c. 8vo. Paris, 1787.

65. Avis aux Habitans des Colonies, particulierement a ceux de l'Isle S. Domingue, sur les principales causes des maladies qu'on y eprouve le plus communément, & sur les moyens de les prevenir. Par *J. F. Lafosse*, Docteur en Medecine de l'Université de Montpellier, Correspondant de la Société Royale de Medecine. 8vo. Paris, 1787.

66. Disser-

66. Differtation & Obfervations fur la Gangrene des Hopitaux, avec les moyens de la prevenir & de la combattre. Par *André Duffaufoy*, Chirurgien en chef du Grand Hotel Dieu de Lyon. 8vo. Geneve, 1787.

67. Traité des Bandages Herniaires dans lequel on trouve, independamment des bandages ordinaires, des machines propres à remedier aux chûtes de la Matrice & du Rectum, à fervir de recipient dans le cas d'anus artificiel, d'incontinence d'urine, &c. Par M. *Juville*, Chirurgien Herniaire. 8vo. Paris, 1786.

68. Idées fur les Secours a donner aux pauvres Malades dans une Grande Ville. 8vo. Paris, 1786.

69. Tarantifmo obfervado en Efpana, conque fe prueba el de la Pulla, dudado de algunos, y tratado de otros de fabulofo : y memorias para efcribir la Hiftoria del infecto llamado Tarantula, efectos de fu veneno en el cuerpo humano, y curacion por la múfica con el modo de obrar de efta, y fu aplication como remedio á varias enfermedades. Su Autor Don *Francifco Xavier Cid*, Socio de la Real Sociedad Bafcongada, Academico de la Real Academia Medica Matritenfe, y medico titular del iluftrisimo Dean y Cabildo de la Santa Iglesia de Toledo, Primada de las Espanas, y del Excelentisimo é ilustrisimo Senor Don Francisco Lorenzana, Arzobispo de dicha ciudad. 4to. Toledo, 1787.

70. Descripcion historica de una nueva especie de Corea, ó Baile de San Vito, originada de la picadura de un insecto, que por los fenómenos seguidos á ella se ha creido ser la Tarantula. Enfermedad de que ha adolecido y curado á beneficio de la musica Ambrosio Silvan: narracion de los sintomas con que se ha presentado, y exposicion fiel y circunstanciada del plan curativo que se ha practicado. Informe a la Real Junta de Hospitales, por el Doctor Don *Bartoleme Pinera y Siles*, Academico de la Real Academia Medica de Madrid, Medico en esta Corte, y uno de los del numero de los Reales Hospitales General y de la Pasion de ella. 4to. Madrid, 1787.

71. Fenomeno raro y singular del primero que se ha conocido entrar en el Hospital General de Madrid por haber sido picado del insecto llamado Tarantula. 4to. Madrid, 1787.

72. Ricerche sulla Materia zuccherina delle sostanzë vegetabili ed animali del S. Dott. *G. M. Savani.* 8vo. Bologna, 1786.

73. Institu-

73. Inſtituzione di Chirurgia di *Giuſeppe Neſſi*, comaſco Dottore in Filoſofia e Medicina, e Prof. d'Oſtetricia e Inſtituz. Chirurg. nella Regia Univerſita di Pavia. Tom. II. 8vo. Pavia, 1786-87.

74. Diſcorſo medico chirurgico intorno alle parotidi che vengono nel corſo delle Febbri acute, del Sig. *Onofrio Valentini*, Profeſſore di Chirurgia del pubblico di Spoleto. 8vo. Perugia, 1786.

75. Sull' uſo del fuoco conſiderato come preſidio Chirurgico. Oſſervazioni pratiche di *Angelo Riboli*, Chirurgo aſtante nel Regio Spedal Maggiore di Milano. 8vo. Milan, 1787.

76. Aminnelſe-Tal ofver Profeſſoren uti Chemien och Pharmaceutiken vid Kongl. Academien i Upſala, ſamt Riddaren af Kongl. Waſa orden, Herr *Torbern Olof Bergman*, hallet for Kongl. Vetenſkaps Academien den 3 Maii, 1786, af deſs Ledamot *Peter Jacob Hjelm*, Proberare. 8vo. Stockholm, 1786.

78. Die Eiſpflanze als ein faſt ſpecifiſches mittel empfohlen. *i. e.* The ice plant recommended as an almoſt-ſpecific remedy. By *John William Frederick Lieb*, M. D. &c. and Phyſician at Mittau. 8vo. Hof, 1785.

N 2 The

The plant here recommended is the *mesembryanthemum cryſtallinum* of Linnæus. The expreſſed juice of it has been found uſeful by the author in bilious and ſpaſmodic complaints, and in diſeaſes of the urinary paſſages. In large doſes it is ſaid to have conſiderable effect as a diuretic.

79. Verſuche ueber das Gehirn und Ruckenmark. *i. e.* Experiments on the Brain and ſpinal Marrow. By *J. Arnemann*, M. D. 8vo. Goettingen, 1787.

80. Beobachtungen aus der Arzneywiſſenſchaft, Chirurgie und Gerichtlichen Arzneykunde; nebſt einer Unterſuchung und Beſchreibung des Quedlinburgiſchen Geſundbrunnens. *i. e.* Obſervations relative to Phyſic, Surgery, and medical Juriſprudence; together with an Inquiry relative to, and Deſcription of, the mineral Waters at Quedlinbourg. By *C. J. A. Ziegler*, M. D. 8vo. Leipſic, 1787.

81. Dr. *John Haygarth*'s Unterſuchung wie den Blattern zuvorzukommen ſey. Aus dem Engliſchen ueberſetzt von Dr. *Joh. F. Ludw. Cappel.* 8vo. Berlin, 1786.

82. *J. Hunter*'s Abhandlung ueber die veneriſche Krankheit. Aus dem Engliſchen. 8vo. Leipſic, 1787.

83. Abhand-

83. Abhandlungen der K. K. Josephinischen medizin. chirurg. Akademie zu Wien. *i. e.* Transactions of the Imperial and Royal Josephine medico-chirurgical Academy at Vienna. Vol. I. 4to. Vienna, 1787.

84. Traité analytique et pratique des eaux thermales d'Ax et d'Ussat, dans le Comté de Foix; avec la description des bains, des douches, des fontaines, et la meilleure maniere de les employer dans les differentes maladies. Par M. *Pilhes*, Med. Intendant de ces Eaux. 8vo. Paris, 1787.

85. Supplement a l'Essai sur les Eaux minerales de Bourbon-l'Archambault en Bourbonnois. Par M. *Faye*, Medecin Intendant desdites Eaux, et pensionné de sa Majesté. 12mo. Paris, 1787.

86. De Vitriolo Albo ejusque usu medico et chirurgico. Auctore *Carolo Henrico Stolte*, Longosalissa - Thuringo. 8vo. Goettingæ, 1787.

87. Dissertatio Inauguralis Medica de Excitantium usu in Febribus potissimum putridis. Auctore *Georgio Henr. Conrado Mehlis*, Goslariensi. 8vo. Goettingæ, 1787.

88. Dissertatio Inauguralis Medica de Angina gina Pectoris vulgo sic dicta. Auctore *Georgio Benjamin Schaeffer*, Hameliensi. 8vo. Goettingæ, 1787.

89. Dissertatio Inauguralis Chemico-Physiologica de Bilis Natura. Auctore *Joanne Fred. Straehl*, Thuno-Helveto. 8vo. Goettingæ, 1787.

90. Dissertatio Physiologico-Medica Inauguralis Animadversiones quædam circa motum Bilis sistens. Auctore *Gulielmo Belcombe*, Anglo. 8vo. Goettingæ, 1787.

91. Dissertatio Medica de Mercurio Tartarisato liquido. Auctore *Joh. Conrad. Theophil. Boelke*, Verda-Hannoverano. 8vo. Goettingæ, 1787.

92. Dissertatio Inauguralis Medica de Nausea ac Vomitu Gravidarum. Auctore *Joann. Friderico Koerber*, Esthono. 8vo. Goettingæ, 1787.

93. Observations sur les Effets des Vapeurs Mephitiques dans l'homme, sur les noyés, sur les Enfans qui paroissent morts en naissant, et sur la Rage; avec un precis du traitement le mieux eprouvé en pareil Cas; sixieme Edition à laquelle on a joint des Observations sur les Effets de plusieurs Poisons dans le Corps de l'Homme, et les Moyens d'en empecher les suites

ſuites funeſtes; par M. *Portal*, Medecin conſultant de Monſieur, Lecteur et Profeſſeur de Medecine au College Royal de France, Profeſſeur adjoint d'Anatomie et de Chirurgie au Jardin du Roi, des Academies des Sciences de Paris, de Boulogne, &c. 8vo. Paris, 1787.

93. Memoires ſur la Neceſſité et les Moyens d'éloigner du milieu de Paris, les tueries des beſtiaux, et les fonderies des ſuifs. 4to. Paris, 1787.

94. Diſſertation ſur le Café, et ſur les moyens propres à prevenir les effets qui reſultent de ſa preparation communement vicieuſe, et à en rendre la boiſſon plus agreable et plus ſalutaire, avec une gravure en taille douce; par M. *Gentil*, Docteur-Regent et ancien Profeſſeur de la Faculté de Medecine en l'Univerſité de Paris; ancien Medecin des Camps et Armées de S. M. le Roi de France. 8vo. 1787.

95. Eſſai ſur l'Hiſtoire Naturelle des fraiſiers; par M. *Duchesne*. 12mo. Paris, 1787.

96. Explication du Syſteme Botanique du Chevalier Von Linné, pour ſervir d'Introduction a l'étude de la Botanique: ouvrage dans lequel on donne, 1°. un precis des ouvrages elementaires de cet auteur; 2°. on examine

ſi ſon

ſi ſon Syſteme eſt le plus ſolidement etabli, ſi l'auteur a été fondé a rejetter tontes les parties de la fleur, et forcé de preferer les organes ſexuels; 3°. on deſigne les ouvrages elementaires et neceſſaires, avec la meilleure maniere de s'en ſervir; 4°. on donne une explication de pluſieurs mots techniques: par M. *Gouan*, Conſeiller, Medecin du Roi, Profeſſeur Royal de Medecine au Ludovicée de Montpellier, aſſocié ordinaire de la Soeieté Royale des Sciences de cette ville, aſſocié honoraire de celle de Florence, &c. 8vo. Montpellier, 1787.

97. Abhandlung ueber dic Nutzbarkeit der Kayſerlichen freyen Reichſtadt Achen, befindlichen Mineialwaſſer, worin angezeigt wird, mit welchem Vortheile ſelbige in verſchiedenen Faellen gebraucht zu werden pflegen, mit mehr als hundert merkwurdigen Krankengeſchichten erlaeutert. *i. e.* A Treatiſe on the Utility of the Mineral Water of the Imperial free City of Aix la Chapelle, in which are ſhewn the Advantages that may be derived from it in different Caſes; the whole being illuſtrated by more than an hundred remarkable Caſes. By *Ferdinand Michels*, Phyſician at Juliers. 8vo. Cologn, 1787.

